# Endometrioid endometrial adenocarcinoma in a premenopausal woman with multiple organ metastases

**DOI:** 10.4103/0971-5851.60053

**Published:** 2009

**Authors:** Nirmala Srikantia, Rekha B., Rajeev A. G., Suman N. Kalyan

**Affiliations:** *Department of Radiotherapy, M S Ramaiah Medical College and Hospital, MSRIT Post, Bangalore - 560085*

**Keywords:** *Adjuvant chemotherapy*, *endometrial carcinoma*, *multiple organ metastases*

## Abstract

Endometrial adenocarcinoma is the third common malignancy of the female genital tract occurring most often in the postmenopausal age group. High tumor grade, advanced surgical stage, and lymphovascular space invasion are implicated as poor prognostic factors for dissemination of disease. We present an unusual case of endometrial adenocarcinoma in a premenopausal woman with simultaneous metastases in brain, liver, skin and skeletal system, within one month of completion of treatment. The role of adjuvant/concurrent chemotherapy in addition to radiotherapy in high risk cases is discussed along with the review of literature.

## INTRODUCTION

Worldwide, endometrial cancer ranks third among genital malignancy in women after cervix and ovary, constituting six per cent of new cancer cases in women and accounting to three per cent of all cancer deaths in women. Approximately 41,200 new cases of endometrial cancer were estimated in 2006 with 7,350 deaths due to the disease according to American Cancer Society Statistics.[1] The most common histological type is Endometrioid adenocarcinoma, constituting 75-80%. The average age at diagnosis is 60 years; disease in women younger than 40 years constitute five per cent. High tumor grade, advanced surgical stage, and lymphovascular space invasion are implicated as poor prognostic factors for dissemination of disease. In the literature metastasis to scalp, skeletal system and brain, lung and liver have been reported in individual patients.[2–4] We report a case of endometroid carcinoma of endometrium in a young premenopausal woman with simultaneous metastases in liver, brain, bone and skin for rarity of presentation.

## CASE REPORT

**Figure 1:**
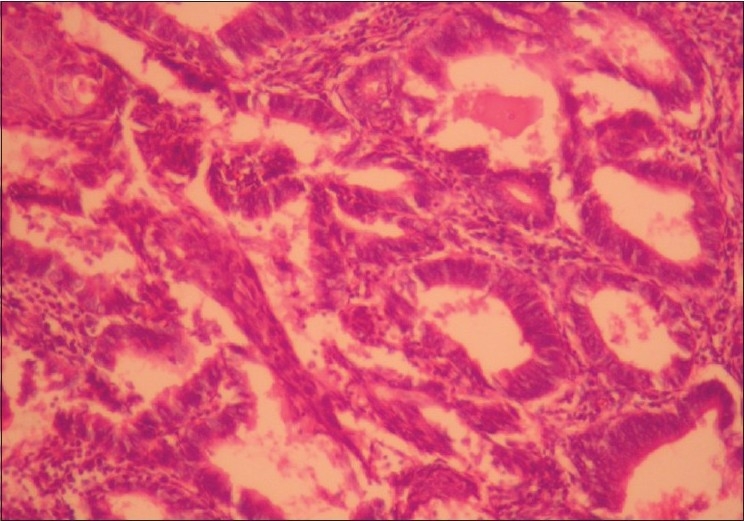
Histopathology of the endometrium (magnification: ×20) showing the closely packed irregular glands lined by stratified columnar epithelium with focal morule formation is seen. Stroma is scanty

**Figure 2:**
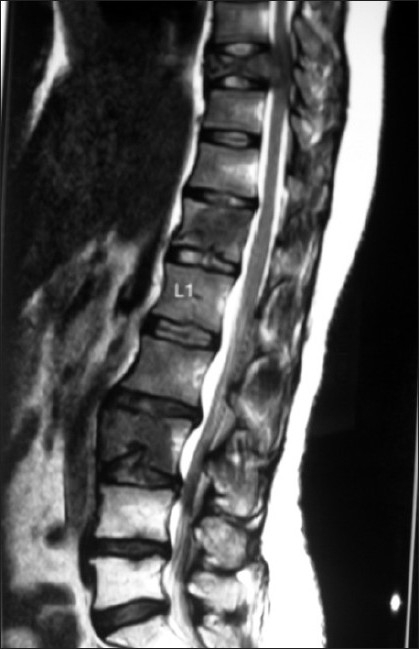
MRI of spine showing the collapse of dorsal 9 vertebra with cord compression

**Figure 3:**
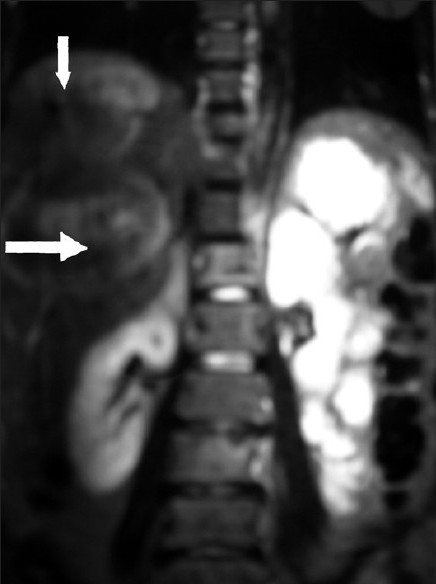
MRI of abdomen showing large space occupying lesions in liver

**Figure 4:**
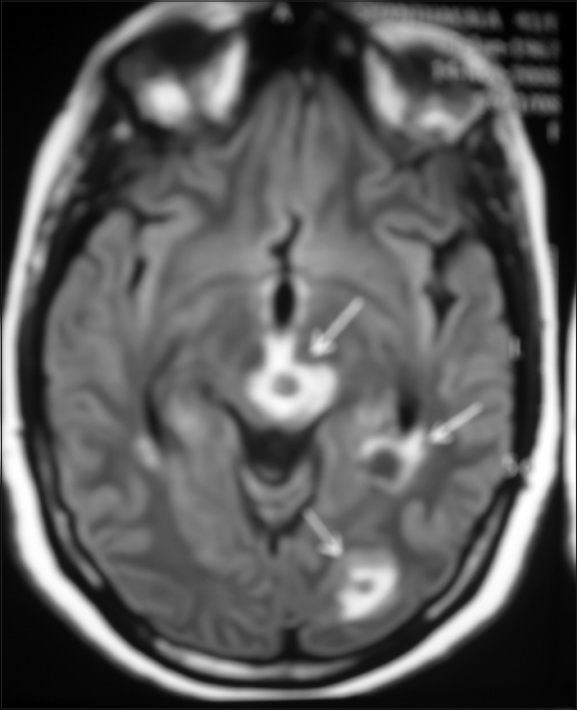
T2 weighted image of MRI brain showing multiple metastatic lesions

**Figure 5:**
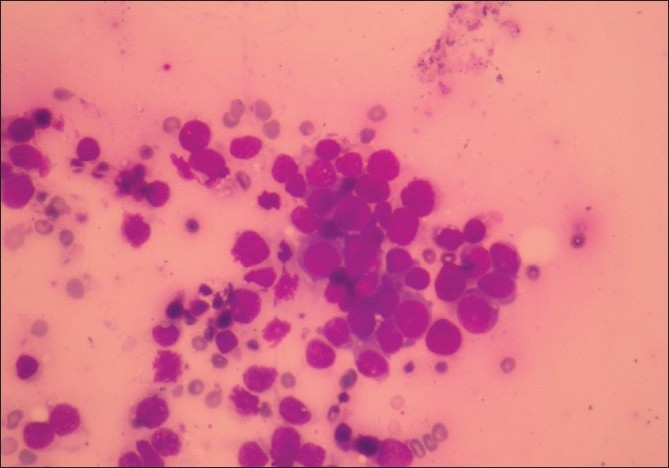
Smear from FNAC of subcutaneous lesions showing loosely cohesive groups of atypical epithelial cells with large dark staining nucleus suggestive of malignancy. MGG stain, ×10

A 41-year-old premenopausal lady diagnosed as FIGO Stage Ic grade III endometroid adenocarcinoma [Figure 1] of uterus was referred for adjuvant radiotherapy after wertheims hysterectomy. Hormone receptor study was negative. She received adjuvant whole pelvis RT and vaginal brachytherapy as per NCCN practice guidelines V.1.2008. One month after treatment, she presented with weakness of lower limbs, fatigue, giddiness and visual complaints of one week duration. MRI of the spine revealed metastases to thoracolumbar vertebrae with cord compression. [Figure 2] and multiple liver metastases [Figure 3]. MRI of brain showed multiple brain metastases [Figure 4]. Subcutaneous lesions were found on scalp and infra axillary region. Fine needle aspiration cytology confirmed them to be metastatic [Figure 5]. She was treated with palliative radiotherapy to spine and brain.

As pelvic disease was under control, she was considered for systemic chemotherapy with paclitaxel and carboplatin. But she developed persistent thrombocytopaenia and leucopenia. Hence bone marrow infiltration was thought of and CT was deferred. She was advised supportive and symptomatic treatment.

## DISCUSSION

Cancer of the endometrium ranks third among genital malignancy in women after cervix and ovary. The most common histological type is Endometrioid adenocarcinoma, comprising 75-80%, serous papillary carcinoma 5-10% and clear cell carcinoma 3-5%. Serous papillary carcinoma and Clear cell carcinomas are aggressive types.[5]

The average age at diagnosis is 60 years. Women diagnosed with endometrial cancer younger than 40 years make up only five per cent. They invariably have specific risk factors such as morbid obesity, chronic anovulation, and hereditary syndromes.

The differentiation of endometrial cancers is one of the most important prognostic factors. Grade 1, 2, and 3 endometrial adenocarcinomas make up 45, 35, and 20% respectively. The five-year survival rate of stage I cancer is 94%, 88%, and 79% for grade 1, 2, and 3 tumors, respectively. High tumor grade, advanced surgical stage, and lymphovascular space invasion have been implicated as poor prognostic factors for dissemination of disease.[5]

Due to early presentation of disease by vaginal bleeding, 80% of endometrial cancers are diagnosed at an early stage. Surgery and radiation therapy form the main treatment. Total abdominal hysterectomy and bilateral salpingo-oophorectomy with lymph node dissection remains the cornerstone of treatment. In all Stage IC and stage IA and IB with grade 2 or 3 histology, and presence of adverse risk factors like lymphovascular space invasion, advanced age, tumor size, lower uterine segment involvement, vaginal brachytherapy ± pelvic radiotherapy is recommended to reduce the risk of pelvic recurrence.[6] Postoperative radiation therapy may improve local control but not the survival for Stage I endometrial cancer. Systemic chemotherapy is reserved for women with disseminated disease or extrapelvic recurrence. Although the combination of cisplatin plus doxorubicin is commonly used, carboplatin plus paclitaxel represents an efficacious, low-toxicity regimen for advanced or recurrent cases.[7] GOG 184 trial indicated addition of paclitaxel to doxorubicin and cisplatin following surgery and radiation did not increase survival. A randomized phase III trial of doxorubicin/cisplatin/paclitaxel and G-CSF versus carboplatin/paclitaxel in patients with stage III and IV or recurrent endometrial cancer (GOG 209) is currently ongoing.

Cisplatin-adriamycin-cyclophosphamide (CAP) therapy has been used as an effective adjuvant chemotherapy in Japan.[8] In a retrospective review of 170 patients with FIGO stage I and II endometrial carcinoma treated at Niigata University Hospital, the patients were divided into low-risk and high-risk groups based on the number of prognostic factors. Among the high-risk group patients, five-year disease-free survival and overall survival were 88.5% and 95.2% in patients treated with adjuvant chemotherapy, and 50.0% and 62.5% in patients who underwent only surgery (*P* = 0.0150, *P* = 0.0226).This study recommended the use of postoperative adjuvant CAP in high-risk groups to decrease distant failure.[9]

Radiation Therapy Oncology Group (RTOG-9708) conducted a Phase II study to assess the safety and toxicity of chemotherapy when combined with pelvic RT for patients with completely resected high-risk endometrial cancer. They recommend for stage I, grade 3, pelvic RT to a dose of 4500 cGy/25 fractions with concurrent Cisplatin 50 mg/m^2^ on day 1 and day 28 followed by vaginal brachytherapy, followed by Carboplatin AUC 4-5 and Taxol 175 mg/m^2^ every four weeks for four cycles. Currently this is the preferred adjuvant regimen.[10]

A recent study by Gaducci A *et al*. in women with Endometroid Endometrial Adenocarcinoma stage Ib and II who developed hematogenous metastases, the multivariate analysis showed that lymphovascular invasion and deep myometrial invasion (*P* = 0.0345) were independent predictive variables for the risk of distant hematogeneous failure. In patients with these pathological findings they advocated enrolment in randomized trials designed to assess the role of adjuvant chemotherapy alone or combined with sequential and/or concomitant external pelvic irradiation.[11]

A phase III (RTOG 9905) study was designed to compare pelvic RT with pelvic radiation therapy plus chemotherapy. The results of this RCT are awaited and may give a better conclusion on management of similar cases.

Recently, poorly differentiated tumors have been found to overexpress the epidermal growth factor Type II receptor. Anti-HER-2/neu-targeted therapy might be a novel and attractive therapeutic strategy in these patients.[7]

## CONCLUSION

Endometrial carcinoma in younger pre-menopausal women, though rare, can be aggressive enough to cause early metastasis to multiple organs. Adjuvant /concurrent chemotherapy with RT is advisable in high risk cases based on available data.
